# Patterns of Oxygen Pulse Curve in Response to Incremental Exercise in Patients with Chronic Obstructive Pulmonary Disease – An Observational Study

**DOI:** 10.1038/s41598-017-11189-x

**Published:** 2017-09-07

**Authors:** Ming-Lung Chuang, I.-Feng Lin, Shih-Feng Huang, Meng-Jer Hsieh

**Affiliations:** 1 Division of Pulmonary Medicine and Department of Internal Medicine, Chung Shan Medical University Hospital, Taichung, 40201 Taiwan, ROC; 2 School of Medicine, Chung Shan Medical University, Taichung, 40201 Taiwan, ROC; 3 Institute and Department of Public Health, National Yang Ming University, Taipei, Taiwan, ROC; 4 Department of Pulmonary and Critical Care Medicine, Chiayi Chang-Gung Memorial Hospital, Chang-Gung Medical Foundation, Chiayi, Taiwan; 5 Division of Respiratory Therapy, Chang Gung University, Taoyuan, Taiwan

## Abstract

In COPD, pulmonary hyperinflation causes decreased stroke volume thereby decreased oxygen pulse (O_2_P). While O_2_P flattening is related to myocardial ischemia in cardiac patients, O_2_P patterns have seldom been explored in COPD. The aims of the study were to investigate O_2_P-curve patterns and associated factors in COPD. Seventy-five patients with stable COPD were enrolled. The demographics, cardiac size, physiological measurements and stress EKG were compared among O_2_P-curve pattern groups. An algorithm to identify O_2_P-curve patterns was developed in 28 patients. In the remaining 45 patients after excluding two with poor effort, this algorithm revealed 20 (44%) flattening, 16 (36%) increasing, and nine (20%) decreasing patterns. The flattening-type group had lower body mass, cardiac size, and diffusing capacity, and larger lung volumes (*p* = 0.05–<0.0001) compared to the increasing-type group. During exercise, the flattening-type group had a lower operable O_2_P and more hyperventilation and dyspnea (*p* = 0.02–<0.01). None had ST-T changes. Most differences were related to body mass and mildly to inspiratory fraction. The decreasing-type group performed higher effort than the increasing-type group (*p* < 0.05). In conclusion, O_2_P flattening was common and was associated with reduced body mass and pulmonary hyperinflation rather than with myocardial ischemia. The decreasing-type may be caused by motivation to exercise.

## Introduction

In non-invasive cardiopulmonary exercise testing (CPET), oxygen pulse (O_2_P) is defined as oxygen uptake ($\dot{{\rm{V}}}$O_2_) divided by heart rate. It indicates the capability of oxygen consumption in all body tissues per heart beat, and is a function of stroke volume and oxygen extraction by cells. O_2_P reflects stroke volume, and is approximately five times the slope of the normal oxygen consumption-heart rate relationship^1^, as oxygen extraction by muscle cells is expected to increase in a predicted manner when exercising^2, 3^. Hence, O_2_P can be continuously monitored non-invasively to reflect stroke volume during CPET.

Reduced O_2_P with a plateau, or decreasing pattern^4, 5^ when approaching peak exercise has been attributed to cardiac dysfunction or myocardial ischemia in patients with cardiac impairment. Although the relationship between a flattened O_2_P curve and severity of myocardial ischemia is not linear^6^, O_2_P remains an indicator of training effect^7^, and a prognosticator of heart failure^8–11^ and primary pulmonary hypertension^12^. Recently, three patterns of O_2_P curve have been reported, two of which include plateaus or decreases which are thought to be related to cardiac dysfunction^4^. Moreover, an O_2_P value ≤80% of the predicted value has been reported to be non-specific for low stroke volume, because anemia, carboxyhemoglobinemia, poor blood oxygenation in the lung, right to left shunt, and low peripheral oxygen extraction have been associated with low O_2_P^3^.

A reduced O_2_P has been reported in patients with chronic obstructive pulmonary disease (COPD), and this has been associated with swings in intrathoracic pressure resulting from deranged ventilatory mechanics^13^ or dynamic hyperinflation^14^. This reduction in O_2_P has been shown to be partly reversed in patients with emphysema after pharmacological^15^ or surgical lung volume reduction^16–18^. To the best of our knowledge, the O_2_P curve patterns in patients with COPD performing CPET has yet to be investigated, given COPD frequently coexists with ischemic heart disease^19^. We hypothesized that O_2_P curve patterns are not related to myocardial ischemia in patients with COPD. The aims of the study, therefore, were to investigate: (1) the types of O_2_P patterns and (2) their associated factors in patients with COPD. These findings may affect the currently-used algorithm for reporting CPET and provide insight into the mechanisms that determine O_2_P curve patterns.

## Methods

### Study Design

In this observational cross-sectional comparative study, we grouped patients based on O_2_P curve patterns obtained from CPET. Due to the variability in O_2_P curves, each curve was smoothed using computer software. Demographics, cardiac size measured with chest radiography and two-dimensional echocardiography, lung function, CPET, and arterial blood gas were compared among groups. The Institutional Review Board of Chung Shan Medical University Hospital (CS11144) and Chang Gung Memorial Hospital (CMRP 443) approved this study and all participants provided written informed consent. The study was conducted in compliance with the Declaration of Helsinki.

### Subjects

The COPD was diagnosed according to the GOLD criteria^20^. The enrollment criteria were patients with COPD who underwent lung function tests and the ratio of their forced expiratory volume in one second (FEV_1_) and forced vital capacity (FVC) was <70%. They were invited to perform the symptom-limited incremental CPET with arterial blood gas and lactate measurements. All patients must be clinically stable, and their medications must be not significantly changed one month before undergoing the tests. The exclusion criteria were if the patients had significant co-morbidities such as left ventricular failure (ejection fraction <50%), atrial fibrillation, renal failure (creatinine >2 mg/dL), cancer, hemoglobin ≤10 g/dL, peripheral artery occlusive disease, and uncontrolled diabetes mellitus or hypertension. Peripheral artery occlusive disease was diagnosed by history, medical record, and symptomatology. Participating in any physical training program during the study period was not allowed.

### Protocols and Measurements

#### Anthropometric and biochemical measurements

Body mass index, triceps skinfold thickness, and mid-upper arm circumference were measured. All measurements were made in triplicate by an experienced nutritionist, and the middle value was recorded for analysis. Complete blood cell analysis, carboxyhemoglobin, and selected biochemical tests were performed.

*Oxygen–cost diagram* (OCD) A 10-cm long vertical line marked with everyday activities was used by the patients to assess daily activities^21^. The distance from point zero was measured and scored.

#### Pulmonary function testing

Air flows and lung volumes were measured by spirometry and plethysmography (6200 Autobox DL, Yorba Linda, CA, USA or MasterScreen™ Body, Carefusion, Wuerzburg, Germany) at body temperature, ambient atmospheric pressure, and fully saturated, using the best of three readings^22–24^. The single-breath technique was used to measure the diffusing capacity for carbon monoxide (D_L_CO). A 12-second maneuver of rapid and deep breathing was used to calculate the maximum voluntary ventilation (MVV). All lung volume data were obtained before inhaling 400 μg of fenoterol HCl and spirometry data were obtained before and after inhaling fenoterol. Maximal inspiratory/expiratory pressures were measured at residual volume and total lung capacity, respectively, before and 7 minutes after exercising (RPM, Micro Medical, Rochester, UK) three times, with a one-minute recovery period between efforts, with the best results being used for analysis.

#### Maximum cardiopulmonary exercise testing

After acclimating to a computer-controlled brake cycle ergometer and revealing stable exercise gas exchange (Medical Graphics, St. Paul, MN, USA), each subject completed a 2 minutes of rest and 2 minutes of unloaded cycling followed by a ramp-pattern exercise test to the symptom limited. Work rate was selected at a rate of 5–20 watts/minute based on a derived protocol formula according to the OCD scores^25^. The $\dot{{\rm{V}}}$O_2_ (ml/min), CO_2_ output ($\dot{{\rm{V}}}$CO_2_) (ml/min), minute ventilation ($\dot{{\rm{V}}}$_E_), pulse rate and oxyhemoglobin saturation (S_P_O_2_), and 12-lead electrocardiography were continuously measured. Blood pressure was measured at the end of each minute and at the point where the patients expressed peak exercise. Dyspnea was scored using the modified Borg scale every minute when the patients were performing the exercise. Calibrations of pneumotachograph and O_2_ and CO_2_ analyzers, anaerobic threshold (AT) measurement, and $\dot{{\rm{V}}}$O_2peak_ predictions were performed as reported previously^25^. $\dot{{\rm{V}}}$O_2peak_ was symptom-limited, and defined as the highest recorded value averaged over the last 15 seconds of loaded exercise.

A pre-requisite for final analysis of the data was the subjects must achieve the maximum exercise effort^26, 27^, including heart rate ≥85% of predicted maximum, respiratory exchange ratio ≥1.09, pH ≤7.35, bicarbonate concentration ([HCO_3_^−^]) ≤21 meq/L, changes (Δ) in [HCO_3_^−^] or [lactate] between at rest and peak exercise ≥4 meq/L. Each criterion represented one point. Each maximum effort level point was scored from 1–6, with the total score representing the effort level of exercise.

1 $$\begin{array}{c}{\rm{Cardiovascular}}\,{\rm{stress}}\,{\rm{level}}\,{\rm{or}}\,{\rm{exercise}}\,{\rm{intensity}}\,{\rm{was}}\,{\rm{defined}}\,{\rm{as}}\,{\rm{heart}}\,{\rm{rate}}\\ {\rm{at}}\,{\rm{peak}}\,{\rm{exercise}}/{\rm{heart}}\,{\rm{rate}}\,{\rm{predicted}}\,{\rm{maximum}},\end{array}$$

where predicted maximum heart rate = 220 − age

2 $${{\rm{O}}}_{2}{{\rm{P}}}_{{\rm{peak}}} \% \,{\rm{predicted}}={{\rm{O}}}_{2}{{\rm{P}}}_{{\rm{peak}}}/{{\rm{O}}}_{2}{{\rm{P}}}_{{\rm{\max }}}{\rm{predicted}}$$

where O_2_P_peak_ = measured O_2_P at peak exercise and O_2_P_max_ predicted = $\dot{{\rm{V}}}$O_2_ predicted maximum/predicted maximum heart rate

The definition of ventilatory limitation was breathing reserve (BR) either <30% or <11–15 L/min, and was calculated as^27^:

3 $${\rm{BR}}=1-{\dot{{\rm{V}}}}_{{\rm{E}}{\rm{peak}}}/{\rm{direct}}\,{\rm{MVV}},$$

where $\dot{{\rm{V}}}$_E peak_/direct MVV expressing $\dot{{\rm{V}}}$_E_ demand/capacity ratio

4 $${\rm{Mean}}\,{\rm{inspiratory}}\,{\rm{tidal}}\,{\rm{flow}}={\rm{tidal}}\,{\rm{volume}}\,({{\rm{V}}}_{{\rm{T}}})\,({\rm{liters}})/{\rm{inspiratory}}\,{\rm{time}}\,(\sec )$$

5 $${\rm{Rapid}}\,{\rm{shallow}}\,{\rm{breathing}}\,{\rm{index}}={\rm{breathing}}\,{\rm{frequency}}\,({\rm{breath}}/\,{\rm{\min }})/{{\rm{V}}}_{{\rm{T}}}\,({\rm{liters}})$$

6 $${\rm{Inspiratory}}\,{\rm{duty}}\,{\rm{cycle}}={\rm{inspiratory}}\,{\rm{time}}/{\rm{total}}\,{\rm{time}}\,{\rm{of}}\,{\rm{breathing}}\,{\rm{cycle}}$$

#### Development of smoothing techniques for O_2_P curve

To avoid breath-by-breath noise, all O_2_P data from unloading to peak exercise were obtained after averaging every 15 seconds with smoothing (Supplement file) using the computer software (Microcal Origin v 4.1, Microcal Software Inc., Northampton, MA, USA). By demonstrating residuals to the model fits, the process of curves smoothing using computer software was attested not causing impact on results (Supplement file: Appendix Figure 3). After developing the smoothing technique, the investigators used the algorithm, which showed intra- and inter-rater agreements using κ statistics of 0.7 (95%CI 0.52–0.89) and 0.7 (95%CI 0.52–0.88), respectively.

#### Chest radiography

Chest radiographs with posteroanterior view were obtained within 1 month from enrollment and evaluated by two pulmonologists blinded to the clinical information. The hila-thoracic ratio, cardiothoracic ratio, and diameter of the anterior descending pulmonary artery on upright posteroanterior chest radiographs were measured^28^ using the DICOM viewing software (Infinitt PACS, v3.0.11.3, VN3, Infinitt, Korea). The inter-rater agreement using Pearson’s correlations were 0.66, 0.87, and 0.58 for the hila-thoracic ratio, cardiothoracic ratio, and diameter of the anterior descending pulmonary artery, respectively (p < 0.01–0.0001). Average values were recorded for analysis.

#### Two-dimensional echocardiography

Two-dimensional echocardiography (iE33, Philips, Seattle, USA) was performed with parasternal, apical and subcostal views^29–31^ within 4 weeks before or after CPET. If there were acute exacerbations of COPD in the time between the two tests, one of the tests was postponed. The echocardiography was conducted by an experienced technician or cardiologist who was blinded to the clinical data, lung function and CPET reports. The stored data were reviewed by two experienced cardiologists who were not blinded to the measurements.

#### Arterial blood sampling and lactate determination

Blood samples were drawn from the brachial artery via an arterial catheter connected to a pressure transducer within the last 15 seconds of each minute after the start of exercise to peak exercise. Plasma lactate was also analyzed (YSI, Yellow Springs, Ohio, USA). The V_D_/V_T_ was calculated as follows:

7 $${{\rm{V}}}_{{\rm{D}}}/{{\rm{V}}}_{{\rm{T}}}=1-0.863\times {\dot{{\rm{V}}}\mathrm{CO}}_{2}/({\dot{{\rm{V}}}}_{{\rm{E}}}\times {{\rm{P}}}_{{\rm{a}}}{{\rm{CO}}}_{2})$$

where V_D_/V_T_ indicates dead space volume and tidal volume ratio. The breathing valve dead space was so small (approximately 30 mL) that it was ignored.

### Statistical Analysis

Data were summarized as mean ± standard deviation or frequency and percentage. All of the data were shown to be normally distributed by the Kolmogorov-Smirnov test and therefore p values were calculated by ANOVA with Tukey’s correction for multiple comparisons to compare means between the three groups. Fisher’s exact method with Holm’s correction for multiple comparisons was used in contingency table analysis for categorical variables. A *p* < 0.05 was considered to be significant, and 0.05–0.1 as marginally significant^32^. Statistical analyses were performed using SAS software v9.4 (SAS Institute Inc., Cary, NC, USA) and Microcal Origin v4.1.

## Results

**Figure 1:**
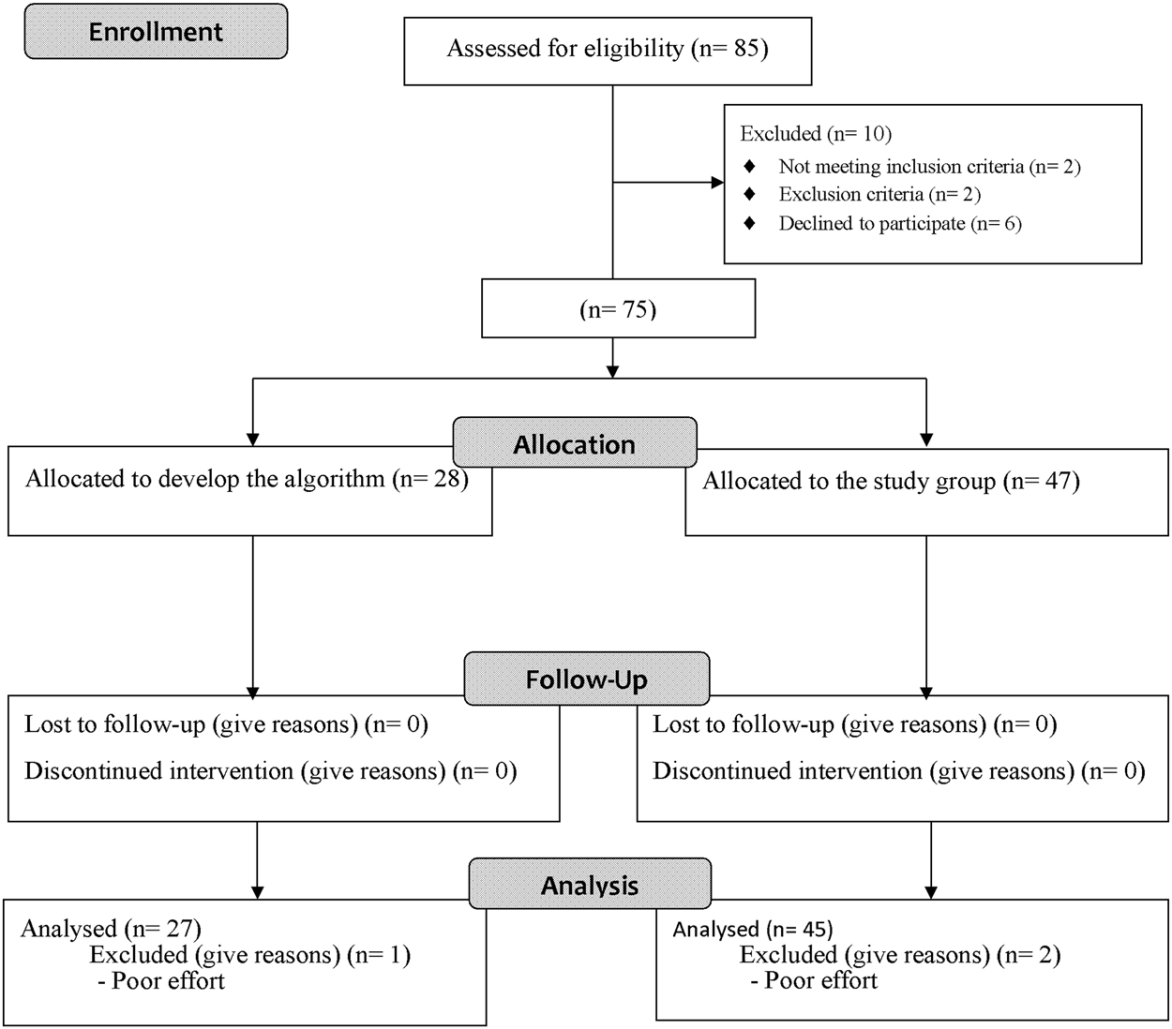
Flow diagram. A total of 85 patients with chronic obstructive pulmonary disease were screened. 75 patients were enrolled and 28 patients of them were used for developing the algorithm of identifying O_2_P-curve patterns. The remaining 47 patients completed the observational study.

Seventy-five patients were enrolled and completed the study (Fig. 1), of whom 28 were used to develop the algorithm to identify O_2_P-curve patterns (Supplement file). The remaining 47 patients were used as the study group, two of whom were excluded from analysis due to poor effort (exercise duration of 2 minutes) leaving 45 patients for the final analysis. Twenty patients (44%) with an increasing O_2_P pattern initially followed by a flattening pattern were classified in the plateau-type group, 16 patients (36%) with an increasing pattern during loaded exercise were classified in the increasing-type group, and nine patients (20%) with a decreasing pattern during the last few minutes were classified in the decreasing-type group.

The plateau-type group had lower body weight, body mass index, and triceps skin-fold compared to the increasing-type group (Table 1, *p* = 0.03–<0.0001) and also lower creatinine levels compared to the decreasing-type group (*p* = 0.05). Differences in other anthropometric and biochemical data were insignificant between the groups. The cardiothoracic ratio in chest radiography and end-diastolic right ventricle area measured in sub-costal 4-chamber view were or tended to be smaller in the plateau-type group (Table 2, *p* = 0.004 and 0.06, respectively).

**Table 1:** Demographic and biochemistry data of patients with chronic obstructive pulmonary disease grouped by the patterns of oxygen pulse (n = 47*, mean ± SD).

N=	Increasing (Inc)	Plateau (Plat)	Decreasing (Dec)	P		
	16	20	9	Inc vs Plat	Plat vs Dec	Inc vs Dec
Age, years	65.7 ± 6.3	65.3 ± 6.7	64.2 ± 2.3	0.98	0.89	0.82
Height, cm	164.6 ± 6.2	165.5 ± 7.2	164.4 ± 5.8	0.90	0.92	1.0
Weight, kg	**67**.**2** ± 9.9	55.3 ± 8.3	60.3 ± 13.8	**0**.**003**	0.44	0.24
Body mass index, kg/m^2^	**24**.**8** ± 3.2	20.1 ± 2.0	22.2 ± 3.9	**<0**.**0001**	0.20	**0**.**09**
Smoke, pack·year	46.6 ± 24.0	39.7 ± 11.6	40.7 ± 24.9	0.54	0.99	0.75
Oxygen-cost diagram, cm.	7.0 ± 1.7	7.1 ± 1.3	6.9 ± 1.1	0.98	0.96	0.99
Triceps skin-fold thickness, mm	**7**.**6** ± 2.9	5.4 ± 1.8	6.1 ± 3.0	**0**.**03**	0.79	0.33
Mid-arm circumference, cm	28.1 ± 4.5	26.3 ± 2.2	28.0 ± 3.3	0.28	0.44	1.0
Hemoglobin, gm	15.0 ± 1.5	14.6 ± 1.7	15.1 ± 1.0	0.68	0.76	1.0
Carboxy-hemoglobin, %	1.3 ± 1.1	1.5 ± 0.8	1.1 ± 0.6	0.75	0.50	0.88
Albumin, g/dL	4.2 ± 0.4	4.1 ± 0.4	4.2 ± 0.3	0.48	0.72	0.98
Creatinine, mg/dL	1.1 ± 0.2	1.0 ± 0.1	**1**.**2** ± 0.2	0.79	**0**.**05**	0.17

*2 patients excluded from the analysis due to poor exercise performance. Bolded numbers indicating statistical significance or the largest number of each variable across the three groups.

**Table 2:** Chest radiographic and 2-dimensional echocardiographic data of patients with chronic obstructive pulmonary disease grouped by the patterns of oxygen pulse (mean ± SD).

N=	Increasing (Inc)	Plateau (Plat)	Decreasing (Dec)	P		
	16	20	9	Inc vs Plat	Plat vs Dec	Inc vs Dec
Chest radiography						
Hilum-thorax ratio	0.36 ± 0.03	0.36 ± 0.04	0.37 ± 0.02	0.91	0.81	0.64
Cardiothoracic ratio	**0**.**49** ± 0.05	0.42 ± 0.06	0.42 ± 0.06	**0**.**004**	0.95	**0**.**06**
Anterior descending PA	1.7 ± 0.4	1.6 ± 0.3	1.6 ± 0.4	0.96	0.95	0.86
2- dimensional echocardiography						
Apical 4-chamber view						
End-diastolic RV, cm^2^	14.8 ± 4.3	12.5 ± 2.9	13.0 ± 3.8	0.18	0.94	0.47
End-systolic RV, cm^2^	8.3 ± 1.9	7.8 ± 1.8	6.9 ± 1.7	0.73	0.44	0.16
Subcostal 4-chamber view, yes/no						
End-diastolic RV, cm^2^	**15**.**6** ± 3.8	13.0 ± 2.8	14.1 ± 3.0	**0**.**06**	0.68	0.50
End-systolic RV, cm^2^	8.3 ± 2.4	7.3 ± 1.8	7.0 ± 2.1	0.36	0.96	0.30
Long and short axes view						
RV wall thickness, mm	6.1 ±2.3	6.1 ±1.7	5.9 ± 2.3	1.0	0.98	1.0

PA: pulmonary artery; RV, right ventricular; IVS: intraventricular septum. Bolded numbers indicating statistical significance or the largest number of each variable across the three groups.

The plateau-type group had a larger TLC and higher rate of inspiratory capacity/TLC ratio (inspiratory fraction) ≤25% (Table 3, all *p* = 0.05–0.01), lower D_L_CO (*p* < 0.0001) and poorer recovery of maximum inspiratory pressure after exercise (*p* = 0.05).

**Table 3:** Lung function of the patients with chronic obstructive pulmonary disease grouped by the patterns of oxygen pulse (mean ± SD).

N=	Increasing (Inc)	Plateau (Plat)	Decreasing (Dec)	P		
	16	20	9	Inc vs. Plat	Plat vs. Dec	Inc vs. Dec
Total lung capacity (TLC), L	6.27 ± 0.90	**6**.**94** ± 1.1	6.00 ± 0.80	0.11	**0**.**05**	0.79
TLC, pred %	128 ± 20	**145** ± 18	123 ± 18	**0**.**02**	**0**.**01**	0.82
Functional residual capacity (FRC), L	4.49 ± 0.75	**5**.**22** ± 1.19	4.36 ± 0.62	**0**.**07**	**0**.**08**	0.94
Residual volume (RV), L	3.52 ± 0.62	4.11 ± 1.10	3.40 ± 0.54	0.11	0.10	0.94
Inspiratory capacity/TLC < 25%,n/subtotal n	3/16	**12**/20	2/9	**0**.**01**	**0**.**06**	0.83
Diffusing capacity for carbon monoxide, pred %	**85** ± 19	57 ± 17	68 ± 5	**<0**.**0001**	0.27	**0**.**06**
Forced vital capacity (FVC), pred %	76 ± 22	88 ± 17	75 ± 23	0.19	0.24	0.98
Forced expired volume in one second (FEV_1_), pred%	49 ± 14	54 ± 22	46 ± 18	0.63	0.56	0.96
Stage 1, n=	0	3	0			
Stage 2, n=	7	7	4	0.73^F^	0.73^F^	
Stage 3, n=	7	8	3			
Stage 4, n=	2	2	2			
FEV_1_/FVC	0.52 ± 0.12	0.48 ± 0.16	0.47 ± 0.07	0.66	0.98	0.66
Maximum inspiratory pressure (MIP), cm H_2_O	73 ± 21	67 ± 16	65 ± 18	0.64	0.96	0.58
Maximum expiratory pressure (MEP), cm H_2_O	**113** ± 29	95 ± 13	103 ± 21	**0**.**07**	0.69	0.51
ΔMIP post-pre exercise, cm H_2_O	**14** ± 19	0 ± 9	5 ± 18	**0**.**05**	0.74	0.33
ΔMEP post-pre exercise, cm H_2_O	5 ± 21	3 ± 16	−3 ± 11	0.92	0.67	0.49

D_L_CO: diffusing capacity for carbon monoxide; pred: predicted; Δ: difference. Bolded numbers indicating statistical significance or the largest number of each variable across the three groups. The p value with a superscript F indicating a Fisher’s test for testing the association between Stages of COPD and the patterns.

**Figure 2:**
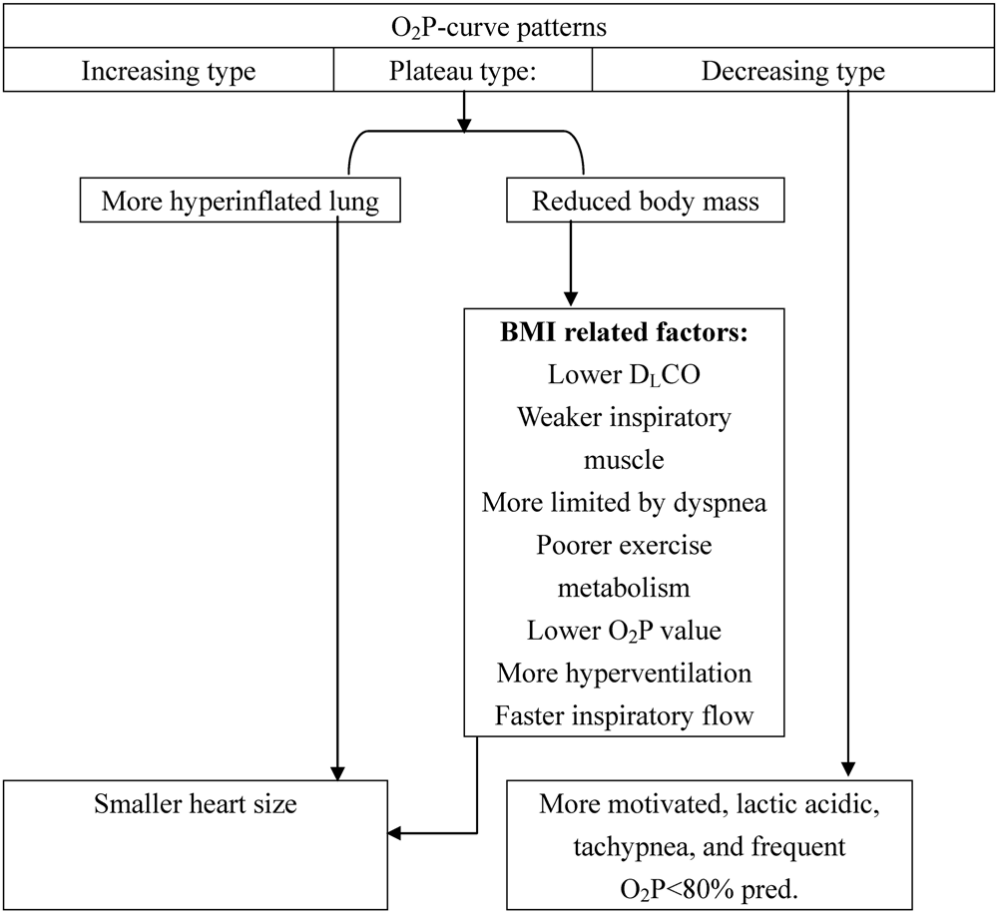
Summary of oxygen pulse (O_2_P)-curve patterns and its relation to demographics and physiology in patients with chronic obstructive pulmonary disease. BMI: body mass index, D_L_CO: diffusing capacity for carbon monoxide.

The frequency of dyspnea limiting exercise was higher in the plateau-type group compared to the increasing-type and decreasing-type groups (Table 4, both *p* < 0.05). Only one patient had chest pain (at the right side). During exercise, none of the patients had ST changes or T wave inversion on EKG. At peak exercise, the plateau-type group had lower $\dot{{\rm{V}}}$O_2_, O_2_P, operable O_2_P (Table 4, all *p* = 0.01–0.02), and higher inspiratory duty cycle, mean inspiratory flow normalized by $\dot{{\rm{V}}}$O_2peak_, $\dot{{\rm{V}}}$_E_/$\dot{{\rm{V}}}$O_2_ and $\dot{{\rm{V}}}$_E_/$\dot{{\rm{V}}}$CO_2_ (all *p* = 0.03–0.0004). The decreasing-type group was similar to the plateau-type group regarding all exercise variables. However, compared to the increasing-type group, the decreasing-type group had a larger decrease in pH (*p* = 0.05), increase in plasma lactate level, maximum effort score, breathing frequency, and rapid shallow breathing index between at rest and peak exercise (all *p* = 0.01–0.04). All of these differences were more related to BMI than to predicted TLC% or the frequency of inspiratory fraction ≤25% (Tables 5 and 6, Fig. 2).

**Table 4:** Cardiopulmonary exercise test and arterial blood gas and lactate data at peak exercise in the patients with chronic obstructive pulmonary disease grouped by the oxygen pulse (O_2_P)-curve patterns (mean ± SD).

Type	Increasing (Inc)	Plateau (Plat)	Decreasing (Dec)	P		
N=	16	20	9	Inc vs Plat	Plat vs Dec	Inc vs Dec
Limiting symptoms						
Dyspnea, n = 34	10(62.5%)	**19**(**95**%)	5(55%)	**0**.**02** ^M^	**0**.**03** ^M^	0.99
Fatigue, n = 12	2(12.5%)	6(30%)	4(44%)	0.26	0.67	0.14
Chest pain on the right side, n=1	0	0	1			
Exercise power, watt	108 ± 46	82 ± 27	95 ± 54	0.16	0.73	0.73
$\dot{{\rm{V}}}$O_2_, mL/min	**1253** ± 369	948 ± 252	1093 ± 403	**0**.**02**	0.52	0.48
$\dot{{\rm{V}}}$O_2_% pred max, %	74 ± 17	68 ± 23	69 ± 23	0.67	0.97	0.88
$\dot{{\rm{V}}}$CO_2_, mL/min	1306 ± 456	1028 ± 316	1204 ± 511	0.12	0.54	0.82
Systolic/diastolic blood pressure, mmHg	226 ± 22/101 ± 13	212 ± 33/98 ± 14	229 ± 45/105 ± 9	0.42/0.70	0.43/0.38	0.98/0.81
Heart rate, beat/min	131.4 ± 22.5	130.8 ± 18.2	144.6 ± 19.0	0.99	0.21	0.27
Heart rate %pred, %	81 ± 12	80 ± 12	88 ± 11	0.97	0.17	0.26
O_2_P, mL/min/beat	**9**.**5** ± 2.2	7.3 ± 1.9	7.5 ± 2.6	**0**.**01**	0.96	**0**.**08**
O_2_P %pred, %	92 ± 18	85 ± 26	79 ± 27	0.66	0.80	0.40
O_2_P < 80%pred, n/subtotal n	4/16	9/20	**6**/9	0.21	0.28	**0**.**04**
ΔO_2_P, peak-rest, mL/min/beat	**6**.**2** ± 1.9	4.4 ± 1.8	4.4 ± 2.2	**0**.**02**	0.99	**0**.**08**
Minute ventilation, L/min	39.4 ± 12.3	38.8 ± 12.2	38.7 ± 13.1	0.99	1.0	0.99
Breathing frequency, b/min	31 ± 4	32 ± 5	**37** ± **8**	0.67	0.12	**0**.**04**
Rapid shallow breathing index, b/L	26.3 ± 8.6	29.3 ± 9.6	**40**.**0** ± 24.2	0.78	0.11	**0**.**04**
Inspiratroy duty cycle	0.4 ± 0.03	**0**.**43** ± **0**.**05**	0.40 ± 0.03	**0**.**03**	0.23	0.88
V_T_/T_I_/$\dot{{\rm{V}}}$O_2_	1.3 ± 0.2	**1**.**6** ± 0.3	1.5 ± 0.3	**0**.**03**	0.75	0.36
Minute ventilation/$\dot{{\rm{V}}}$O_2_	31.6 ± 4.7	**41**.**2** ± 8.5	36.6 ± 6.5	**0**.**0004**	0.24	0.21
Minute ventilation/$\dot{{\rm{V}}}$CO_2_	30.9 ± 5.1	**38**.**2** ± 6.4	34.3 ± 7.7	**0**.**003**	0.27	0.40
Dead space and tidal volume ratio	0.40 ± 0.10	0.45 ± 0.09	0.44 ± 0.13	0.35	0.97	0.63
P_a_CO_2_, mm Hg	**48**.**9** ± 8.1	43.0 ± 7.6	47.3 ± 6.8	**0**.**08**	0.36	0.87
P_a_O_2_, mm Hg	69.1 ± 16.5	73.4 ± 18.6	68.3 ± 14.5	0.75	0.75	0.99
ΔpH, rest-peak	0.07 ± 0.04	0.08 ± 0.02	**0**.**1** ± 0.03	0.43	0.23	**0**.**03**
Lactate, meq/L	4.3 ± 1.6	5.5 ± 1.9	**6**.**5** ± 2.2	0.18	0.38	**0**.**03**
ΔLactate, peak-rest, meq/L	3.2 ± 1.5	4.2 ± 1.8	**5**.**6** ± 2.3	0.17	0.22	**0**.**01**
Maximum points	2 ± 2	3 ± 2	**4** ± 2	0.22	0.43	**0**.**04**
ΔBorg/Δ$\dot{{\rm{V}}}$O_2_, A.U./mL/min	10.0 ± 4.7	14.0 ± 6.5	12.3 ± 9.5	0.18	0.80	0.68

$\dot{{\rm{V}}}$O_2_: oxygen uptake, V_T_/T_I_/$\dot{{\rm{V}}}$O_2_: tidal volume/inspiratory time ratio normalized with $\dot{{\rm{V}}}$O_2_, A.U.: absolute unit. Δ: difference. Maximum points: respiratory exchange ratio ≥1.09, heart rate ≥85% of predicted maximum, pH ≤ 7.35, bicarbonate concentration ([HCO_3_^−^]) ≤21 meq/L, the change in [HCO_3_^−^] between at rest and at peak exercise ≥4 meq/L, and the change in lactate concentration between at rest and at peak exercise ≥4 meq/L. Each criterion represented one point for maximum exercise. The points of maximum effort level were scored from 1–6 points. The accumulated points represented the effort level of exercise. Please refer to text. Bolded numbers indicating statistical significance or the largest number of each variable across the three groups.The p values with a superscript M indicating a Holm’s adjusted *p* value 0.06–0.1 by a Fisher’s test. Other symptoms limited also occurred in the increasing-type group, one being too hot, two having dry mouth, one oxyhemoglobin desaturation; one having foot sliding out on biking in the plateau-type group. Two patients having multiple limiting symptoms in the plateau-type group, one patient having multiple limiting symptoms in the decreasing-type group.

**Table 5:** Body mass index (BMI), inspiratory fraction (IC/TLC), and total lung capacity (TLC)% correlated with the variables significant differences across the three O_2_P patterns (N = 45).

	Correlation Coefficients		
	BMI	IC/TLC	TLC%
Cardiothoracic ratio	0.641^‡^	0.395^*^	−0.497^**^
EDRV, cm^2^	0.399^†^	0.168	−0.216
IC/TLC	0.284^¶^	—	−0.486^†^
TLC%	−0.462^**^	—	—
Diffusing capacity for CO% pred.	0.507^†^	0.364^*^	−0.253^¶^
Maximum expiratory pressure _pre_, cmH_2_O	0.434^**^	0.063	−0.358^*^
Maximum inspiratory pressure _post-pre_, cmH_2_O	0.133	−0.17	0.009
O_2_P_peak_, mL/beat	0.418^**^	0.357^*^	−0.138
O_2_P_peak_, operable, mL/beat	0.427^**^	0.348^*^	−0.149
V_T_/T_I_/$\dot{{\rm{V}}}$O_2 peak_	−0.325^*^	0.001	0.13
Inspiratory duty cycle _peak_	−0.399^**^	0.139	−0.216
Minute ventilation/$\dot{{\rm{V}}}$O_2 peak_	−0.418^**^	0.082	0.21
Minute ventilation/$\dot{{\rm{V}}}$_CO2 peak_	−0.486^†^	−0.16	0.339^*^
Breathing frequency _peak_, breath/min	−0.119	0.214	−0.075
Rapid shallow breathing index _peak_, breath/min/L	−0.231	−0.19	−0.054

EDRV: subcostal 4-chamber view end-diastolic right ventricle area, CO: carbon monoxide, pre: before exercise, post-pre: difference between after and before exercise, V_T_/T_I_/$\dot{{\rm{V}}}$O_2_peak: tidal volume and inspiratory time ratio normalized by oxygen uptake at peak exercise, $\dot{{\rm{V}}}$CO_2_: CO_2_ output. ^*^ *p* < 0.05, ^**^<0.01, ^†^<0.001, ^‡^<0.0001, ^¶^<0.1.

**Table 6:** Multiple linear regression with body mass index (BMI) and inspiratory fraction (inspiratory capacity and total lung capacity ratio) on the variables significant different across three oxygen pulse (O_2_P) patterns (N = 45).

	BMI		IC/TLC	
	coefficient	SE	coefficient	SE
Cardiothoracic ratio	0.011^†^	0.003	0.213^*^	0.101
EDRV, cm^2^	0.395^*^	0.16	2.479	6.54
Diffusing capacity for carbon monoxide % pred	0.028^**^	0.008	0.287	0.368
Maximum expiratory pressure _pre_¸cm H_2_O	2.962^**^	0.979	−19.8	42.5
O_2_P_peak_, mL/beat	0.228^*^	0.093	7.67^¶^	4.17
O_2_P_peak_, operable, mL/beat	0.207^*^	0.081	6.4^¶^	3.65
O_2_P_peak_% pred	−0.011	0.01	0.836^¶^	0.455
V_T_/T_I_/$\dot{{\rm{V}}}$O_2 peak_	−0.029^*^	0.012	0.378	0.563
Inspiratory duty cycle _peak_	−0.004^*^	0.002	0.125	0.083
Minute ventilation/$\dot{{\rm{V}}}$O_2 peak_	−1.093^**^	0.324	22.3	14.5
Minute ventilation/$\dot{{\rm{V}}}$_CO2 peak_	−0.941^**^	0.275	−1.762	12.3

EDRV: sub-costal 4-chamber view end-diastolic right ventricle area, CO: carbon monoxide, pre: before exercise, post-pre: difference between after and before exercise, V_T_/T_I_/$\dot{{\rm{V}}}$O_2_ peak: tidal volume and inspiratory time ratio normalized by oxygen uptake at peak exercise, $\dot{{\rm{V}}}$CO_2_: CO_2_ output. All variables were standardized. To avoid co-linearity problems, all variables were centered. SE is the standard error of the parameter estimates. ^*^ *p* < 0.05, ^**^<0.01, ^†^<0.0001, ^¶^<0.1.

## Discussion

In this study, we identified three O_2_P curve patterns in response to symptom-limited incremental exercise in patients with COPD: increasing, plateau, and decreasing. To the best of our knowledge, this is the first study to investigate O_2_P curve patterns in patients with COPD.

### O_2_P curve flattening and stress EKG

Detection of exercise-induced myocardial ischemia can be improved by integrating the duration of O_2_P flattening and $\dot{{\rm{V}}}$O_2_ work rate slope with stress EKG in patients with documented coronary artery disease^33^. Adding CPET and gas exchange measurements such as O_2_P curve flattening has been reported to be of value in diagnosing and quantifying both overt and occult myocardial ischemia^5^. However, another study reported that flattening of the O_2_P curve during exercise can only be used to detect extensive but not mild myocardial ischemia^6^. Moreover, a study on multivariate criteria in diagnosing cardiac causes of exercise limitation found that the O_2_P curve flattening pattern was not superior to predicted O_2_P%^4^. Despite these inconsistent clinical implications of O_2_P curve flattening, 16.6% of patients with COPD have ischemic heart disease^34^, which enhances the importance of this pattern. However, we cannot definitively conclude that this pattern was associated with myocardial ischemia in patients with COPD, as none of the patients had significant ST segment or T wave changes in EKG or significant chest pain or oppression during exercise.

### O_2_P curve flattening and its associated factors

The patients with a plateau-type O_2_P curve had a reduced body mass, smaller heart size, weaker inspiratory muscles, lower diffusing capacity, greater lung volume, and higher rate of inspiratory fraction ≤25% (Tables 1–3 and Fig. 2). This group also had a lower metabolism and O_2_P, and a higher frequency of dyspnea and hyperventilation at peak exercise (Table 4). These aforementioned differences among the three groups were most correlated with BMI and less with inspiratory fraction or predicted TLC% (Table 5). O_2_P and operable O_2_P were more significantly associated with BMI than with inspiratory fraction, but insignificantly with predicted TLC% (Tables 5 and 6). There were significant differences in the frequency of inspiratory fraction ≤25% among the three groups, however inspiratory fraction was modestly correlated with O_2_P% _peak_ predicted (Tables 3 and 6). These findings suggest that inspiratory fraction influences the O_2_P pattern in a threshold manner rather than a linear relationship.

The cardiac size was associated with both BMI and inspiratory fraction, with the latter contributing less (Table 6). This is partly consistent with a previous study that suggested that hyper-inflated lungs or emphysema may compress the heart^35^ or reduce O_2_P^14^. We speculate that both reduced body mass and dynamic hyperinflation contribute to intra-thoracic pressure swings which mechanically constrain blood return to the right heart and/or increase afterload to the right and left ventricles^13^ in a threshold manner, thereby flattening the O_2_P curve. The additional importance of inspiratory fraction is that it is associated with mortality^36^ when the fraction is ≤25% or predicts forecasting peak $\dot{{\rm{V}}}$O_2_ < 60% when the fraction is <28%^37^.

Other factors influencing the O_2_P curve patterns might be also co-linear with BMI (Fig. 2). $\dot{{\rm{V}}}$O_2_ and O_2_P at peak exercise were significantly different across the three groups (Table 4, *p* = 0.02 and 0.01, respectively), however the differences became insignificant when peak $\dot{{\rm{V}}}$O_2_ or O_2_P was presented with predicted value, suggesting the differences were co-linear with body mass. This is consistent with previous reports in which $\dot{{\rm{V}}}$O_2_ was affected by body mass^38^, ventilation capacity^16^, dynamic hyperinflation^39^, and dead-space ventilation, and O_2_P at peak exercise was affected by BMI, inspiratory fraction ≤25%, predicted FEV_1_%, and hand grip force^14^. In contrast, the rapid shallow breathing index differed significantly between the decreasing-type and the increasing-type groups (Table 4), however the index was associated with motivation to perform exercise (see below) and not BMI or inspiratory fraction (Table 5).

### Decreasing O_2_P curve pattern

The decreasing-type group was similar to the plateau-type group in demographics, cardiac size, lung function, and exercise physiology except for having higher creatinine levels and lower lung volumes (Tables 1–4, all *p* ≤ 0.05). This was because the plateau-type had smaller muscle mass but larger lung volumes. Compared to the increasing-type, the decreasing-type group had higher maximum effort scores, more acidity, and more rapid breathing pattern at peak exercise, suggesting that this group was more motivated to perform (Table 4).

### Factors dissociated with O_2_P curve patterns

The O_2_P curve patterns were not related to pack-years of cigarette smoking, OCD or forced spirometry values. OCD mimics exercise capacity expressed in MET normalized with body weight^21^, which may weaken the association between OCD and O_2_P curve pattern. The O_2_P curve patterns were not related to exercise power, dynamic heart rate, blood pressure, ventilation capacity, dead space ventilation, P_a_O_2_, or P_a_CO_2_ at peak exercise. In addition, dynamic dead space ventilation was significantly correlated with dynamic O_2_P (with peak O_2_P, r = −0.65, *p* < 0.0001, with operable O_2_P, r = −0.68, *p* < 0.0001), suggesting that dynamic dead space ventilation cannot explain O_2_P curve patterns.

#### Study limitations

First, we did not thoroughly investigate myocardial ischemia by nuclear medicine or coronary angiography, so that the relationship between myocardial ischemia and O_2_P curve flattening could not be established. However, EKG did not reveal significant ST segment or T wave changes, and no chest pain or oppression occurred during exercise (Table 4) and recovery from exercise. Moreover, no cardiac events developed during the follow-up period (at least 2 years). These findings indicate that myocardial ischemia may not have been the major cause of O_2_P curve flattening. Second, selection bias may have occurred as patients with obvious coronary artery disease and left heart failure were excluded, so the results cannot be generalized to all subjects who perform CPET. Third, according to a previous report on co-morbidities with ischemic heart disease^34^, eight of our cohort should have had ischemic heart disease, although we did not find this. Fourth, we did not measure dynamic inspiratory capacity. However, it seems reasonable that dynamic hyperinflation can be deduced from static hyperinflation since both are highly correlated^39^. Fifth, muscle extraction of oxygen has been assumed to be predictable or the frequency of abnormal muscle extraction of oxygen is low in the general population and patients with COPD. However, the rate of mitochondrial myopathy has been estimated to be 8.5% in the general population^40^. Sixth, patient’s BMI or body surface area is related to the cardiovascular size^41^ and may influence the magnification factors of radiography thereby influencing parallax and measurements in chest radiographs^42^. However, the cardiovascular size measured with cardiothoracic and hila-thoracic ratios reported in the current study is less influenced by body size^41^. However, the possibility of impact of the magnification factors on measurement of the anterior descending pulmonary artery cannot be excluded. In addition, we did not evaluate the inter-rater agreement in measurement of cardiac size with two-dimensional echocardiography thereby probably introducing measurement bias. Lastly, because high-resolution computed tomography was not performed to evaluate the severity of emphysema, the relationships among O_2_P curve patterns, emphysema, and inspiratory fraction could not be determined.

#### Clinical implications

By categorization of patients with COPD into the three groups according to the O_2_P curve patterns, the clinicians may acknowledge that O_2_P curve flattening is much less associated with myocardial ischemia thereby preventing performing unnecessary investigations. Accordingly, providing the optimal management to improve the O_2_P curve flattening can be reached by ameliorating body mass and inspiratory fraction. Whether these measurements can be used in a prognostic manner is to be shown. Finally, further research on the O_2_P curve patterns in patients with heart failure and with COPD concurrently and direct measure of hemodynamics of both patient groups is recommended.

## Conclusions

By analyzing O_2_P with a smoothing technique, we identified three patterns of O_2_P curve, and the flattening pattern was common in patients with COPD when performing CPET. This pattern was further related to exercise-limiting dyspnea and reduced body mass, and modestly related to pulmonary hyperinflation but not to myocardial ischemia. The decreasing-type may be caused by motivation to exercise.

## Electronic supplementary material

Developing an algorithm to identify patterns of oxygen pulse
