# Supplementary material for: Patterns of Oxygen Pulse Curve in Response to Incremental Exercise in Patients with Chronic Obstructive Pulmonary Disease – An Observational Study
Source: Sci Rep. 2017 Sep 7;7:10929. doi: 10.1038/s41598-017-11189-x (PMC5589739; doi:10.1038/s41598-017-11189-x)
Supplement: Supplementary file 1 — Developing an algorithm to identify patterns of oxygen pulse [file 41598_2017_11189_MOESM1_ESM.doc]

**Patterns of Oxygen Pulse Curve in Response to Incremental Exercise in Patients with Chronic Obstructive Pulmonary Disease – An Observational Study**

Ming-Lung Chuang, M.D.^1,2^, I-Feng Lin, Dr.P.H.^3^, Shih-Feng Huang, M.D.^1,2^, Meng-Jer Hsieh, M.D.^4,5^

**SUPPLEMENT**

**Developing an algorithm to identify patterns of oxygen pulse**

Before starting to analyze the data, another 28 patients aged 69±10 years, 162±7 cm in height, and 62±9 kg in weight with chronic obstructive pulmonary disease (forced vital capacity, FVC, 72±15% pred., forced expired volume in one second, FEV_1_, 57±17% pred., FEV_1_/FVC 58±10% post-bronchodilator) were enrolled for developing the algorithm of identifying O_2_P-curve patterns. One patient was excluded from analysis due to poor effort (loaded exercise duration 2 minutes). All the O_2_P data from rest to the peak watts of exercise for each individual were retrieved after averaging every 15 seconds using the manufacture’s software. For visual inspection of the O_2_P-curve patterns, the increasing-type was achievement of peak O_2_P at peak exercise as the patient exercised (Appendix figure 1, left panel); the plateau-type, achievement of peak O_2_P before peak exercise followed by a checkpoint where a plateau developed as the patient continued to exercise (middle panel); the decreasing-type, achievement of peak O_2_P before peak exercise followed by a direct drop in O_2_P or followed by a plateau then a drop (right panel).[^5^](#_ENREF_5) As relatively noisy O_2_P data were encountered despite 15-sec average being done, to determine the checkpoint or its change after the checkpoint might be difficult for some cases (Appendix figure 2, left panels). To overcome the difficulties, the whole patients’ O_2_P data were then smoothed by the adjacent averaging smoothing of five points (Microcal Origin v 4.1, Microcal Software Inc., Northampton, MA, USA) named as the computer assisted method (Right panels, red thick lines). The O_2_P-curve patterns were determined on the computer with the Microcal program and defined by change of the cursor moved along the smoothed curve. Increasing-type was defined as the cursor moved continuously increasingly along the curve during the loaded exercise. The percent change of O_2_P during the last two minutes of loaded exercise was calculated (Appendix table). The plateau-type was defined as the cursor moved along the curve during the loaded exercise revealing a checkpoint after which the cursor did not move up and down. The percent change of O_2_P between the checkpoint and peak of loaded exercise was calculated. The decreasing-type was defined as the cursor moved along the curve during the loaded exercise revealing a checkpoint after which the cursor immediately moved down or reached plateau then moved down. The percent change of O_2_P between the checkpoint and peak of loaded exercise was calculated.

To attest the process of curves smoothing using computer software not causing impact on results, residuals to the model fits, denoting the difference between the fitted function and the measured responses were performed (Appendix figure 3). Except for a small negative or positive value of 0.1 during the first approximate 4 minutes after start of loaded exercise, the residuals are less than 1% for the remainder of loaded exercise. The results denote that the goodness of model fits was excellent.

**Supplement figure 1. V**isual method for three representatives of the oxygen pulse (O_2_P)-curve patterns in response to incremental exercise in patients with chronic obstructive pulmonary disease. Left panel: increasing pattern, middle panel: plateau pattern, right panel: decreasing pattern. For details, please refer to Appendix. Straight arrows indicating work load, curved arrow indicating the checkpoint for plateau-type or decreasing-type.

**Supplement figure 2.** Three representatives of having difficulties in judging the oxygen pulse (O_2_P)-curve patterns in response to incremental exercise in patients with chronic obstructive pulmonary disease. Panels A and C: difficulties in judging the oxygen pulse (O_2_P)-curve patterns by visual method. Panels B and D: data from panels A and C, respectively, smoothed with adjacent averaging method (red thick lines). Panel B becoming clear to be an increasing pattern, panels D becoming clear to be a plateau pattern, respectively. Solid lines indicating work load. Arrows indicating start of unloaded exercise.

**Supplement figure 3.** Upper panel shows the excellent fit of the function (solid line) for O_2_ pulse (solid line with open circle symbol) from a representative. Dashed line indicates watts of exercise. Lower panel shows except for a small negative or positive value of 0.1 during the first approximate 4 minutes after start of loaded exercise, the residuals are less than 1% for the remainder of loaded exercise.

Supplement Table. N=27, mean±SD

|  | Increasing type | Plateau type | Decreasing type |
| --- | --- | --- | --- |
| Visual method, n= | 10 | 14 | 3 |
| Computer assited method, n= | 12 | 14 | 1 |
| Duration of change in oxygen pulse, min | 2±0 | 1.9±0.8 |  |
| Change in oxygen pulse | 10.7±4.6% | 0.4±1.4%* | -4.10% |

*p < 0.0001 when compared between increasing type and plateau type.
